# Multivariate Analysis of Inflammatory and Regenerative Mediators in an In Vitro Canine Osteoarthritis System Treated with Platelet Gel Supernatants

**DOI:** 10.3390/gels12070615

**Published:** 2026-07-09

**Authors:** Jorge U. Carmona, Catalina López

**Affiliations:** 1 Grupo de Investigación Terapia Regenerativa, Departamento de Salud Animal, Universidad de Caldas, Calle 65 No. 26-10, Manizales 170004, Colombia; 2 Grupo de Investigación Patología Clínica Veterinaria, Departamento de Salud Animal, Universidad de Caldas, Calle 65 No. 26-10, Manizales 170004, Colombia

**Keywords:** dog, degenerative joint disease, platelet-rich plasma, orthobiologic gel, principal component analysis

## Abstract

Osteoarthritis (OA) is characterized by interactions among inflammatory and extracellular matrix mediators that may not be fully captured through individual evaluation. This study investigated the multivariate organization and temporal dynamics of selected mediators in a lipopolysaccharide-challenged in vitro canine OA cartilage–synovium explant coculture system treated with platelet-rich gel supernatant (PRGS) and platelet-poor gel supernatant (PPGS). Tissue explants obtained from six dogs were exposed to PRGS or PPGS at 25% and 50% concentrations. Interleukin-1β (IL-1β), IL-10, transforming growth factor beta 1, platelet-derived growth factor BB, and hyaluronic acid were quantified at 1 and 48 h. Principal component analyses (PCA) were performed using both individual mediator concentrations and biologically relevant mediator ratios, which were intended to capture the balance among inflammatory, regulatory, GF-related, and matrix-associated responses beyond absolute mediator concentrations alone, and principal component scores were evaluated using linear mixed-effects models. Mediator-based PCA revealed that variability was distributed across multiple dimensions, with significant treatment, time, and treatment × time effects detected in different principal components. Ratio-based PCA concentrated a larger proportion of total variance within the first two components (81.1%) than mediator-based PCA (61.4%) and identified treatment- and time-related effects involving regulatory and matrix-associated mediator balances. Ratio-derived PCA showed greater structural stability across resampling analyses than the mediator-based approach. PRGS 50% generated the most pronounced multivariate separation, particularly within components associated with GF- and matrix-related mediator profiles. These findings indicate that biologically relevant mediator ratios provide complementary information regarding the organization of inflammatory and regenerative responses in this experimental system.

## 1. Introduction

Osteoarthritis (OA) is characterized by a complex and dynamic microenvironment in which inflammatory cytokines and growth factors (GFs) coexist and interact within the joint [1,2]. Rather than being driven by a single mediator, OA progression reflects the coordinated and dynamically regulated activity of pro-inflammatory signals, anti-inflammatory modulators, anabolic pathways, and mechanometabolic programs that collectively shape joint homeostasis and its failure [3]. This limitation has increasingly motivated the application of multivariate analytical approaches to complex biological datasets [4,5,6].

In vitro systems of OA provide controlled platforms to investigate early inflammatory and regulatory mechanisms [7]. Lipopolysaccharide (LPS) stimulation is widely used to reproduce key inflammatory pathways relevant to OA, including induction of interleukin 1-beta (IL-1β) and modulation of other cytokines and GFs, such as IL-10, transforming growth factor beta-1 (TGF-β1), platelet-derived growth factor BB (PDGF-BB), and hyaluronic acid (HA) [8,9]. However, when such mediators are evaluated individually, results may appear heterogeneous or even contradictory, particularly in systems exposed to biologically active hemocomponents [10].

In addition to inflammatory activation, orthobiologic blood-derived products such as platelet-rich plasma (PRP) have been increasingly explored as modulators of joint inflammation and tissue repair in OA [11,12]. Following activation, PRP undergoes gel formation and releases a complex array of cytokines, GF, and matrix-associated mediators into the surrounding milieu [13,14]. The resulting platelet-rich gel supernatant (PRGS) represents the bioactive soluble fraction released after activation of PRP, while activation of platelet-poor plasma (PPP) yields platelet-poor gel supernatant (PPGS). These cell-free hemocomponents contain multiple bioactive mediators whose biological effects may be better understood using multivariate analytical approaches [15,16].

Comparative evaluation of orthobiologic hemocomponents has traditionally relied on individual mediator concentrations or isolated biological readouts. However, because PRGS and PPGS differ in the composition and relative balance of bioactive mediators released after activation, their biological effects may be more appropriately characterized using multivariate approaches that evaluate coordinated relationships among mediators rather than absolute concentrations alone.

Mechanistically, LPS binds to Toll-like receptor 4 (TLR4), activating downstream nuclear factor kappa B (NF-κB)-dependent transcriptional programs that promote the expression of catabolic cytokines and matrix-degrading enzymes [17,18,19]. This pathway is not only a convenient experimental trigger but is also increasingly recognized as biologically relevant in OA [20,21]. Growing evidence links LPS-related signaling and metabolic endotoxemia to low-grade inflammatory processes associated with synovial activation and cartilage degradation [21,22,23]. Therefore, LPS-challenged in vitro systems may represent a biologically relevant platform for investigating inflammatory and regulatory mediator responses in OA-related environments [20,21].

Within this framework, the mediators selected in the present study were chosen to represent complementary biological domains of joint homeostasis. IL-1β was included as a central pro-inflammatory cytokine associated with cartilage catabolism and synovial activation [24,25]. IL-10 was selected as a regulatory cytokine capable of limiting excessive inflammatory responses [26,27]. TGF-β1 and PDGF-BB were examined as GFs involved in cellular proliferation, matrix remodeling, and repair-related signaling [28,29,30]. Hyaluronic acid (HA), a major structural component of synovial fluid and extracellular matrix, was assessed as a marker of matrix-associated responses and synovial activity. Collectively, these mediators encompass inflammatory, regulatory, growth factor-related, and matrix-associated aspects of OA biology, providing a biologically relevant framework for evaluating coordinated mediator responses [4,5].

Emerging evidence in inflammatory biology indicates that cytokines and GFs interact through complex and interrelated biological processes rather than isolated linear pathways [3,31,32]. Therefore, a multivariate framework may better capture the structure and temporal evolution of these mediators in experimental OA systems [15,16]. By examining relationships among multiple mediators simultaneously, these approaches provide information that complements analyses based on individual biomarkers [33].

Moreover, biologically meaningful ratios may capture relationships among mediators that are not evident from absolute concentrations alone. Because inflammatory, regulatory, and GF-related responses often depend on the balance among multiple mediators, analysis of mediator ratios may provide complementary information to that obtained from individual biomarker concentrations. Therefore, multivariate analyses based on both individual mediator concentrations and biologically relevant ratios may offer a more comprehensive characterization of mediator organization within the experimental system.

The present study aimed to characterize the multivariate organization and temporal dynamics of cytokines and GF in an in vitro system of osteoarthritis challenged with LPS and treated with distinct PRP hemoderivatives. Specifically, we sought to: (1) evaluate the temporal dynamics of key inflammatory and growth mediators; (2) assess their interrelationships and coordinated behavior using complementary multivariate analyses based on both individual mediator concentrations and biologically meaningful mediator ratios; (3) determine whether PRGS and PPGS induce distinct multivariate mediator profiles in a dose-dependent manner; and (4) evaluate whether multivariate structures derived from mediator ratios provide complementary information to those derived from individual mediator concentrations.

To our knowledge, multivariate approaches integrating both individual mediator concentrations and biologically relevant mediator ratios have not been previously applied to this canine OA coculture model. Because inflammatory and regenerative responses emerge from coordinated interactions among multiple mediators, evaluating their relationships may provide biologically relevant information that is not apparent from isolated biomarker measurements. Consequently, PCA was used to identify dominant patterns of variation and to characterize the multivariate organization of mediator responses within the experimental system.

We hypothesized that cytokine and growth factor responses would exhibit distinct multivariate structures when analyzed using individual mediator concentrations and biologically relevant mediator ratios. We further hypothesized that ratio-based analyses would capture treatment- and time-related variation differently from analyses based on individual mediators.

## 2. Results and Discussion

### 2.1. Baseline Cellular and Molecular Characteristics of the Hemocomponents

Cellular characteristics are summarized in Table 1. PRP showed platelet enrichment relative to whole blood (1.45-fold) with concomitant WBC reduction, whereas PPP demonstrated marked depletion of both cell types. These findings confirm effective fractionation prior to supernatant preparation. Baseline molecular profiles of PRGS and PPGS are presented in Table 2. All mediators were detectable in both hemocomponents. PRGS exhibited substantially higher concentrations of TGF-β1 (2.96-fold higher than PPGS) and PDGF-BB (6.42-fold higher than PPGS), whereas IL-1β, IL-10, and HA showed comparable levels between preparations.

The cellular composition of the PRP used in this study was consistent with a pure platelet-rich plasma (P-PRP) or leukocyte-reduced PRP according to the classification proposed by Dohan-Ehrenfest et al. [13], as evidenced by moderate platelet enrichment accompanied by substantial leukocyte depletion relative to whole blood. Following activation with calcium gluconate and incubation for 3 h, both PRGS and PPGS contained detectable concentrations of all evaluated mediators, indicating that both preparations generated bioactive secretomes despite their distinct cellular compositions.

The marked enrichment of TGF-β1 and PDGF-BB observed in PRGS compared with PPGS was expected and is consistent with the higher platelet concentration present in the parent PRP. Both GFs are predominantly stored within platelet α-granules and are released during platelet activation [34]. In contrast, IL-1β, IL-10, and HA showed relatively similar concentrations between preparations, indicating that not all mediators were equally influenced by platelet enrichment. This finding may reflect differences in the biological origin of the evaluated molecules, as some mediators are more closely associated with cellular release during activation, whereas others may be influenced to a greater extent by the baseline plasma composition of the hemocomponents.

### 2.2. Correlation Structure and Time-Dependent Associations

**Figure 1:**
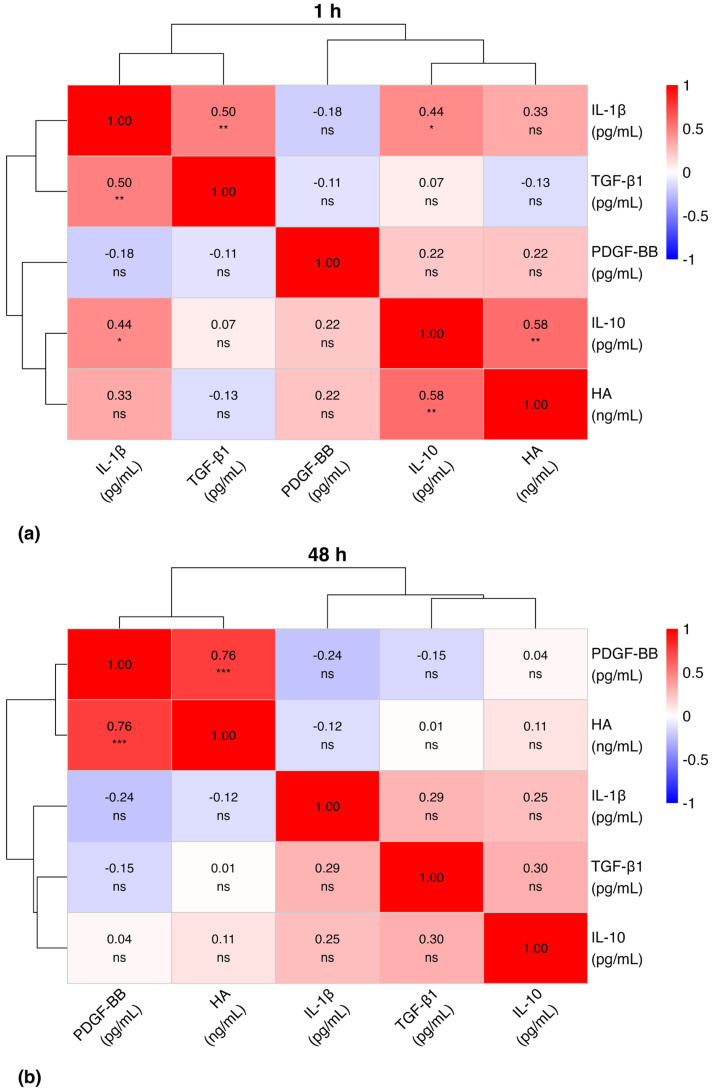
Spearman correlation heatmaps of inflammatory, growth factor-related, and matrix-associated mediators measured in the canine cartilage–synovium coculture system at 1 h (**a**) and 48 h (**b**) following lipopolysaccharide (LPS) challenge (n = 6). Colors represent Spearman correlation coefficients (ρ), ranging from –1 (blue) to +1 (red), and correlation coefficients are displayed within each cell. Statistical significance after Benjamini–Hochberg false discovery rate (FDR) correction is indicated as * adjusted *p* < 0.05, ** adjusted *p* < 0.01, *** adjusted *p* < 0.001; ns, not significant. Dendrograms represent hierarchical clustering based on the correlation coefficients. Abbreviations are as in Table 2.

Spearman correlation matrices stratified by time are presented in Figure 1a,b.

At 1 h, three positive correlations remained significant after false discovery rate (FDR) correction: IL-10 and HA (ρ = 0.576, FDR-adjusted *p* = 0.0019), IL-1β and TGF-β1 (ρ = 0.496, FDR-adjusted *p* = 0.0089), and IL-10 and IL-1β (ρ = 0.444, FDR-adjusted *p* = 0.0197). No other pairwise correlations remained significant following FDR adjustment.

At 48 h, a single positive correlation remained significant after FDR correction between HA and PDGF-BB (ρ = 0.759, FDR-adjusted *p* = 1.27 × 10^−6^). No additional pairwise correlations remained significant after correction for multiple testing.

Hierarchical clustering based on Spearman correlation coefficients is shown in Figure 1a,b.

The correlation analysis revealed a limited number of significant associations after correction for multiple testing, indicating that most mediators behaved relatively independently within the coculture system. Nevertheless, the pattern of significant correlations differed between 1 h and 48 h, suggesting temporal changes in the organization of mediator responses following LPS challenge.

These findings suggest that the relationships among individual mediators changed throughout the experimental period. The temporal differences observed in the correlation structure support the use of multivariate approaches to evaluate patterns among multiple mediators beyond individual biomarker responses [35,36].

### 2.3. Multivariate Organization of Individual Mediator Responses

The first two principal components explained 61.44% of the total variance (PC1: 34.40%; PC2: 27.04%), whereas the first three components accounted for 75.92% of the total variability. The complete five-component solution explained 100% of the variance observed across mediator profiles. The loading structure of the five principal components is summarized in Table 3.

**Figure 2:**
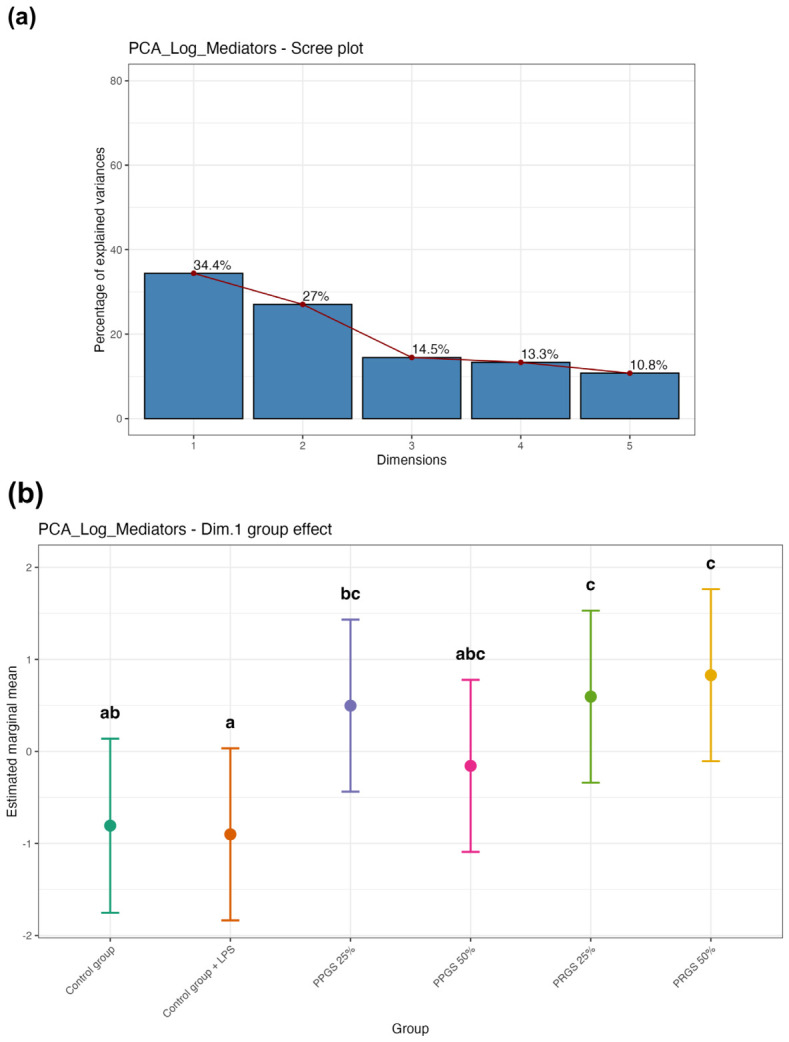
Multivariate organization of mediator responses and treatment-related differences in PC1. Multivariate organization of inflammatory and regenerative mediators in the canine cartilage–synovium coculture system (n = 6). (**a**) Scree plot showing the percentage of variance explained by each principal component (PC) derived from the mediator-based principal component analysis (PCA). (**b**) Estimated marginal means (EMMs) and 95% confidence intervals for PC1 according to treatment group. Different letters indicate significant differences identified by Holm-adjusted pairwise comparisons (*p* < 0.05). Other abbreviations are as in Table 2.

The mediator-based PCA indicated that variability within the coculture system was distributed across multiple dimensions rather than being dominated by a single principal component. Although the first two components explained more than 60% of the total variance, additional dimensions contributed meaningful proportions of variability, supporting retention of the complete five-component solution. This distribution suggests that biologically relevant information was captured across several complementary dimensions of the dataset rather than being concentrated within a single dominant axis (Figure 2a).

The loading structure revealed that inflammatory cytokines, GF, and extracellular matrix-related mediators contributed jointly to the multivariate organization of the system. PC1 was primarily influenced by IL-10, IL-1β, and TGF-β1 and may therefore reflect overall mediator activity, whereas subsequent components captured alternative configurations involving PDGF-BB, HA, and TGF-β1. Notably, PC5 was characterized by opposing contributions of IL-1β and IL-10, suggesting an inflammatory–regulatory balance. Although these biological interpretations should be considered exploratory, they provide a useful framework for understanding the coordinated patterns represented by the principal components [35].

These findings also place the results of the previous univariate analysis of this experimental model into a broader context. In that study [10], IL-1β emerged as one of the most responsive biomarkers and consequently became a central element in the interpretation of treatment-related effects. However, the present multivariate analysis indicates that the biological response was not driven by IL-1β alone, but rather by coordinated changes involving inflammatory, regulatory, GF, and matrix-associated mediators. Similar observations have been reported in multivariate analyses of complex biological systems, where several principal components are often required to adequately represent coordinated variation among interrelated biological variables. Together, these results support the value of complementing traditional univariate approaches with multivariate analyses to better characterize relationships among multiple mediators [36].

### 2.4. Treatment- and Time-Related Modulation of Mediator-Derived Principal Components

Significant treatment effects were detected for PC1, PC2, PC4, and PC5, whereas PC3 was not affected by treatment (Table 4). Time significantly influenced PC1, PC3, and PC5, while no temporal effects were observed for PC2 or PC4. Significant treatment × time interactions were identified for PC4 and PC5, whereas no interaction effects were detected for PC1, PC2, or PC3 (Table 4).

Pairwise Holm-adjusted comparisons indicated that the significant group effect observed for PC1 was primarily driven by differences between PRGS-treated groups and both control conditions (Figure 2b).

**Figure 3:**
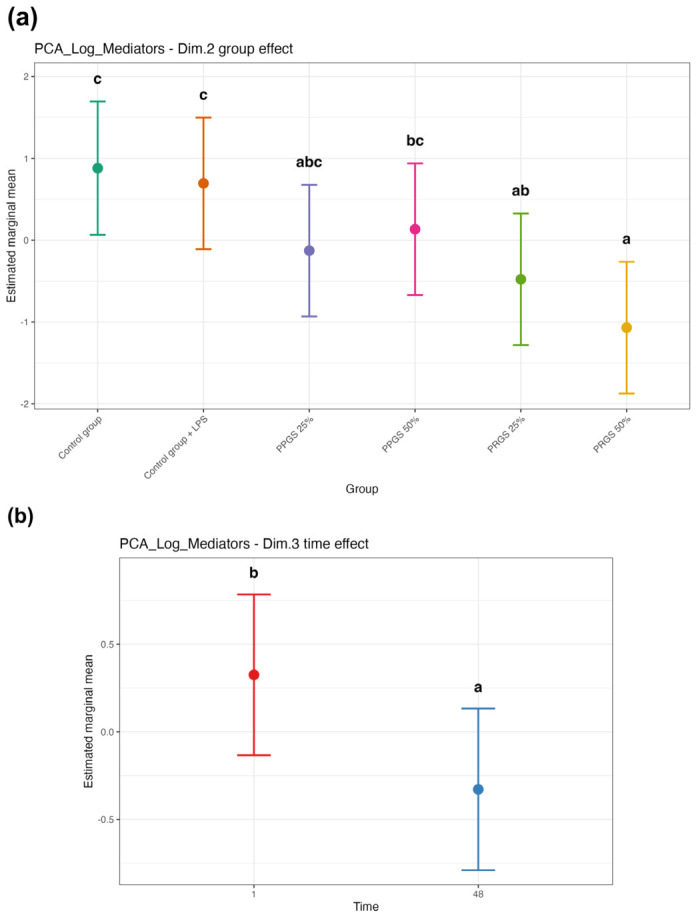
Treatment- and time-related effects on mediator-derived principal components in the canine cartilage–synovium coculture system (n = 6). (**a**) Estimated marginal means (EMMs) and 95% confidence intervals for principal component 2 (PC2) according to treatment group. (**b**) Estimated marginal means (EMMs) and 95% confidence intervals for principal component 3 (PC3) at 1 h and 48 h. Different letters indicate significant differences identified by Holm-adjusted pairwise comparisons (*p* < 0.05). Other abbreviations are as in Table 3.

For PC2, the largest separation was observed for PRGS 50%, which differed from both control groups (Figure 3a). PC3 showed a significant temporal shift between 1 h and 48 h (Figure 3b).

**Figure 4:**
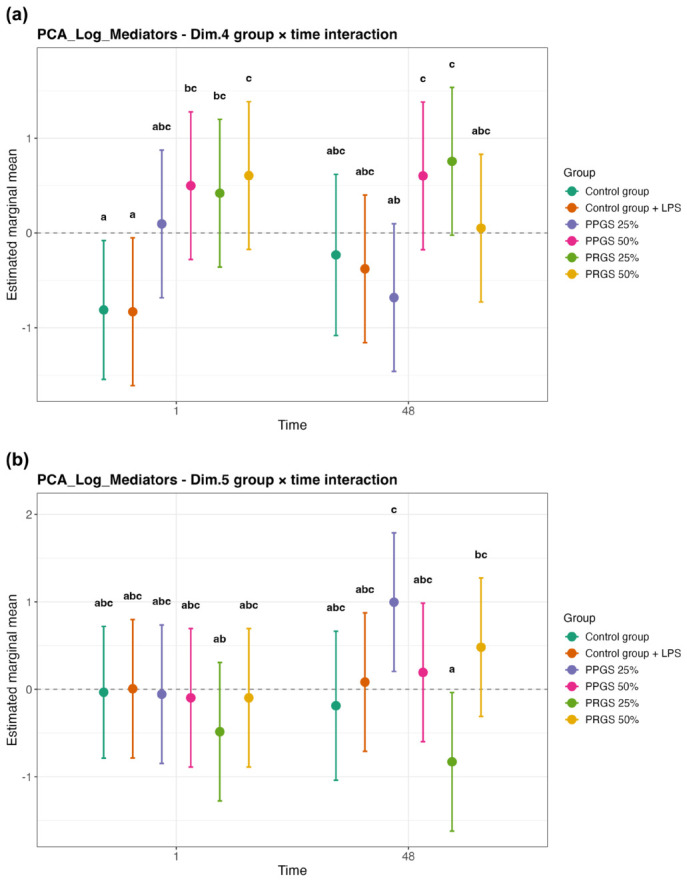
Treatment × time effects on mediator-derived principal components in the canine cartilage–synovium coculture system (n = 6). (**a**) Estimated marginal means (EMMs) and 95% confidence intervals for principal component 4 (PC4) according to treatment group and time point. (**b**) Estimated marginal means (EMMs) and 95% confidence intervals for principal component 5 (PC5) according to treatment group and time point. Different letters indicate significant differences among estimated marginal means identified by Holm-adjusted pairwise comparisons (*p* < 0.05). Other abbreviations are as in Table 3.

In contrast, PC4 and PC5 exhibited treatment-dependent temporal responses, consistent with the significant treatment × time interactions identified by the mixed-effects models (Figure 4a,b).

Significant treatment × time interactions were detected for PC4 and PC5. For PC5, which was characterized by opposite contributions of IL-1β and IL-10, the interaction suggests that the relationship between inflammatory and regulatory mediators varied according to both treatment and time. PC4 also showed treatment-dependent temporal changes, although no overall time effect was detected. Collectively, these results indicate that treatment- and time-related effects were distributed across multiple principal components and involved combinations of mediators rather than a single variable.

### 2.5. Multivariate Organization of Biologically Relevant Mediator Ratios

**Figure 5:**
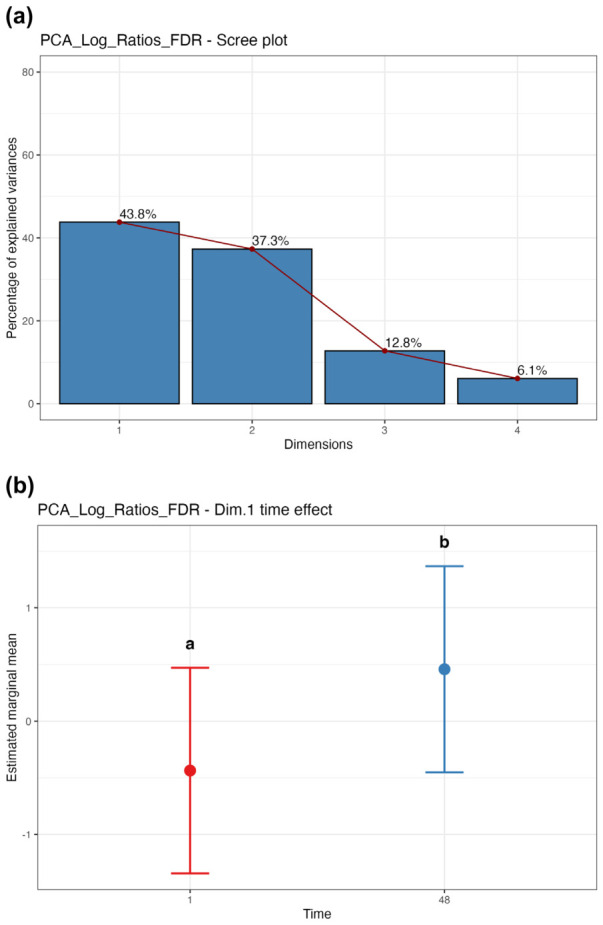
Multivariate organization of biologically relevant mediator ratios in the canine cartilage–synovium coculture system (n = 6). (**a**) Scree plot showing the percentage of variance explained by each principal component (PC) derived from the ratio-based principal component analysis (PCA). (**b**) Estimated marginal means (EMMs) and 95% confidence intervals for PC1 according to time point. Different letters indicate significant differences identified by Holm-adjusted pairwise comparisons (*p* < 0.05). Other abbreviations are as in Table 2.

The first two principal components explained 81.12% of the total variance (PC1: 43.81%; PC2: 37.32%), whereas the first three components accounted for 93.90% of the total variability. The complete four-component solution explained 100% of the variance observed across ratio profiles. The loading structure of the four principal components is summarized in Table 5. The proportion of variance explained by each component is shown in the scree plot (Figure 5a).

In contrast to the mediator-based PCA, a larger proportion of the total variance was concentrated within the first two components of the ratio-based PCA, which together explained more than 80% of the overall variability. This indicates that the relative relationships among mediators were organized along fewer dominant dimensions than those observed when individual mediators were analyzed separately [33,35].

The loading structure revealed a clear separation of the principal components. PC1 was primarily defined by opposite contributions of the HA/IL-10 and PDGF-BB/HA ratios and may therefore be interpreted as a HA–PDGF-BB balance dimension. PC2 was strongly influenced by both IL-10/IL-1β and TGF-β1/IL-1β and may represent an IL-1β-centered regulatory balance. PC3 was also largely determined by these ratios, although with opposite directions of association, suggesting a regulatory mediator contrast between IL-10- and TGF-β1-related signals. In contrast, PC4 showed comparatively smaller and more evenly distributed loadings across variables, precluding a straightforward biological interpretation.

### 2.6. Treatment- and Time-Related Modulation of Ratio-Derived Principal Components

The effects of treatment, time, and treatment × time interaction on the ratio-derived principal components are summarized in Table 6. Significant treatment effects were detected for PC2 and PC3, whereas temporal effects were observed for PC1, PC2, and PC3. A significant treatment × time interaction was identified for PC2, while PC3 showed a trend toward significance. No significant effects were detected for PC4.

**Figure 6:**
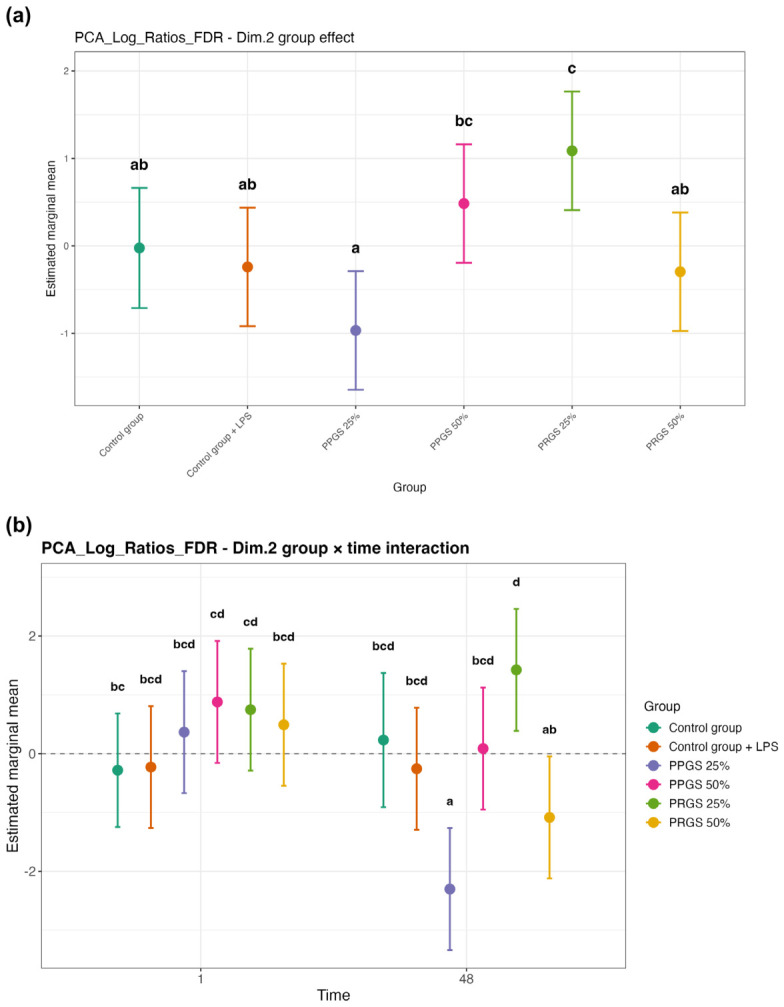
Treatment and treatment × time effects on ratio-derived principal component 2 (PC2) in the canine cartilage–synovium coculture system (n = 6). (**a**) Estimated marginal means (EMMs) and 95% confidence intervals for PC2 according to treatment group. (**b**) Estimated marginal means (EMMs) and 95% confidence intervals for PC2 according to treatment group and time point. Different letters indicate significant differences among estimated marginal means identified by Holm-adjusted pairwise comparisons (*p* < 0.05). Other abbreviations are as in Table 2.

**Figure 7:**
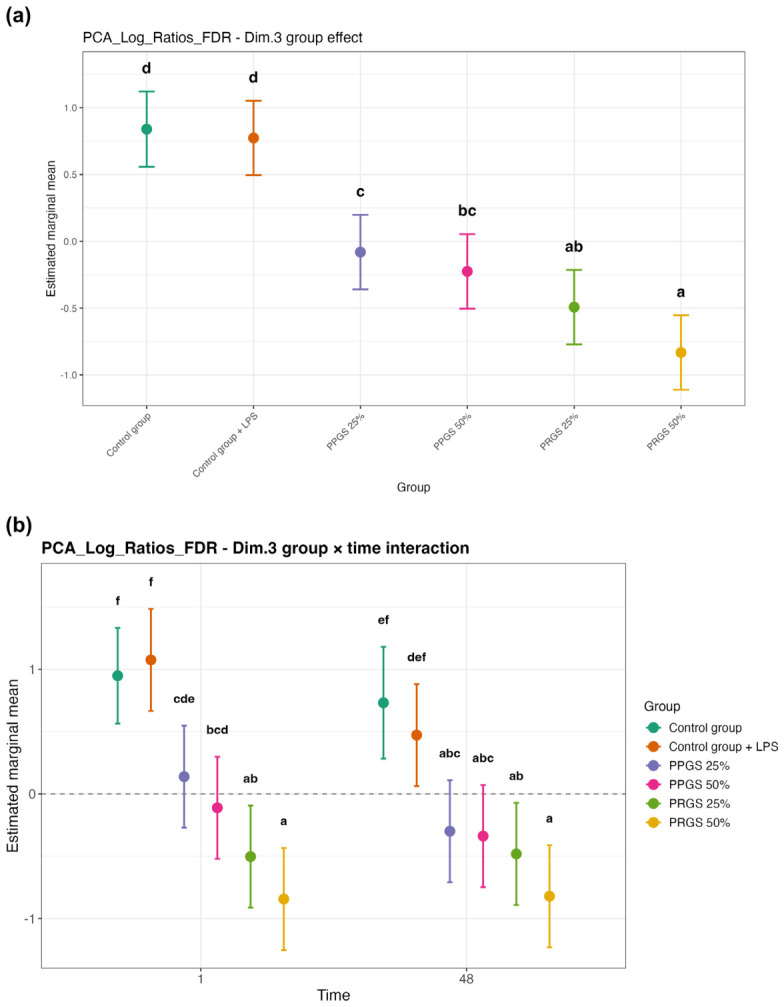
Treatment and treatment × time effects on ratio-derived principal component 3 (PC3) in the canine cartilage–synovium coculture system (n = 6). (**a**) Estimated marginal means (EMMs) and 95% confidence intervals for PC3 according to treatment group. (**b**) Estimated marginal means (EMMs) and 95% confidence intervals for PC3 according to treatment group and time point. Different letters indicate significant differences among estimated marginal means identified by Holm-adjusted pairwise comparisons (*p* < 0.05). Other abbreviations are as in Table 3.

Pairwise Holm-adjusted comparisons indicated a significant temporal shift in PC1 between 1 h and 48 h (Figure 5b). For PC2, significant differences were observed among treatment groups and across time points, consistent with the significant treatment × time interaction identified by the mixed-effects model (Figure 6a,b). PC3 also exhibited significant group differences (Figure 7a), together with differences among treatment groups and time (Figure 7b). No significant pairwise differences were detected for PC4.

The significant interaction suggests that the effects of the different hemocomponents on these regulatory relationships evolved over time rather than remaining constant throughout the incubation period. Notably, PC2 was the only component that simultaneously exhibited significant treatment, time, and treatment × time effects, highlighting its relevance within the ratio-based PCA structure.

Because this component was defined by opposite contributions of the IL-10/IL-1β and TGF-β1/IL-1β ratios, the observed effects indicate that treatment-related variation extended beyond absolute mediator concentrations and involved changes in the relative organization of inflammatory and regulatory signals. In contrast, PC4 showed no significant effects, suggesting that this component contributed little to the treatment- or time-related variation observed in the dataset.

Taken together, these results indicate that biologically relevant mediator ratios captured treatment- and time-dependent variation through a relatively small number of dominant dimensions. Unlike analyses based exclusively on individual mediator concentrations, the ratio-derived PCA emphasized shifts in the relationships among mediators, providing complementary information regarding the organization of inflammatory, regulatory, growth factor-related, and matrix-associated signals within the coculture system.

This observation is consistent with the original univariate analysis of this canine coculture model, in which treatment effects were primarily interpreted through changes in individual mediators such as IL-1β [10], and with subsequent multivariate analyses performed in an equine tendon explant model, where principal component analysis similarly identified coordinated mediator patterns that were not fully apparent from individual biomarker responses [36].

### 2.7. Stability and Robustness of Multivariate Signatures

#### 2.7.1. Bootstrap Assessment of PCA Stability

For the mediator-based PCA, several loading coefficients exhibited broad bootstrap confidence intervals, with multiple intervals crossing zero across PCs 1–5. For the ratio-based PCA, the principal loadings defining PCs 1–3 exhibited bootstrap confidence intervals that did not cross zero. For ratio-derived PC1, the HA/IL-10 and PDGF-BB/HA ratios displayed loading intervals of 0.75–0.95 and −0.95 to −0.71, respectively, with sign stability values of 1.00. For ratio-derived PC2, the IL-10/IL-1β and TGF-β1/IL-1β ratios exhibited loading intervals of 0.56–0.88 and 0.57–0.89, respectively, with sign stability values of 1.00. Ratio-derived PC3 was characterized by loading intervals of 0.38–0.58 for IL-10/IL-1β and −0.59 to −0.39 for TGF-β1/IL-1β, both with sign stability values of 1.00. Complete bootstrap statistics are provided in Table S1.

#### 2.7.2. Leave-One-Dog-Out Assessment of Treatment-Related Effects

For the mediator-based PCA, group effects were retained in 67%, 100%, 50%, 100%, and 83% of leave-one-dog-out iterations for PCs 1–5, respectively. Time effects were retained in 83%, 33%, 100%, 0%, and 33% of iterations for PCs 1–5, respectively. Group-by-time interaction effects were retained in 0%, 33%, 17%, 67%, and 33% of iterations for PCs 1–5, respectively.

For the ratio-based PCA, group effects were retained in 33%, 100%, 100%, and 0% of iterations for PCs 1–4, respectively. Time effects were retained in 83%, 100%, 100%, and 17% of iterations for PCs 1–4, respectively. Group-by-time interaction effects were retained in 17%, 83%, 17%, and 0% of iterations for PCs 1–4, respectively. Correlations between original and leave-one-dog-out scores ranged from 0.741 to 0.986 for mediator-derived principal components and from 0.864 to 0.995 for ratio-derived principal components. Complete leave-one-dog-out statistics are provided in Tables S2 and S3.

The stability analyses supported the robustness of the multivariate findings. Several mediator-derived components exhibited broad bootstrap confidence intervals, whereas the principal loadings defining ratio-derived PCs 1–3 remained stable across bootstrap iterations, with confidence intervals that did not cross zero and sign stability values of 1.00. Similar patterns were observed in the leave-one-dog-out analyses, where the principal treatment- and time-related effects were generally retained after sequential exclusion of individual donors.

Overall, the ratio-derived PCA exhibited greater stability than the mediator-based PCA, as reflected by narrower bootstrap confidence intervals, higher leave-one-dog-out score correlations, and more consistent retention of significant effects. These findings indicate that biologically relevant mediator ratios provided a robust representation of the multivariate structure observed in the coculture system. Consistent with recommendations for PCA interpretation, the stability analyses support the reproducibility of the principal multivariate patterns identified in this study [33].

PCA has been widely applied in OA research, particularly in gait and biomechanical studies, where it has consistently revealed that disease-related changes are organized along dominant latent axes rather than isolated variables [15,37,38,39]. In these contexts, PCA has demonstrated that complex waveform alterations reflect structured, multidimensional adaptations. However, its application to inflammatory mediator networks in experimental OA systems remains limited. The present findings illustrate the applicability of PCA for summarizing coordinated patterns among inflammatory, regulatory, growth factor-related, and matrix-associated mediators within an experimental OA system.

Importantly, this structural reorganization was not fully captured by individual mediator analysis [10]. Mixed-effects modeling of single biomarkers revealed heterogeneous patterns, whereas PC1 scores derived from the extended dataset detected significant time and group effects. The inclusion of biologically meaningful ratios concentrated a larger proportion of explained variance within the first principal components and facilitated the description of coordinated system behavior [3].

The biologically relevant ratios evaluated in this study were defined a priori based on established relationships among inflammatory, regulatory, GF-related, and matrix-associated mediators rather than selected through data-driven optimization. Their value lies in representing biologically plausible balances among complementary biological processes, allowing coordinated mediator responses to be explored within a multivariate framework rather than through isolated biomarker concentrations alone.

Notably, some of the multivariate patterns identified in the present study were not readily apparent from individual mediator analyses alone. Although PRGS exhibited baseline enrichment of TGF-β1 and PDGF-BB, treatment-related effects within the coculture system were distributed across multiple principal components and biologically relevant mediator ratios. These findings suggest that coordinated mediator relationships may provide complementary information to absolute biomarker concentrations when evaluating orthobiologic responses [3,31,32].

Unlike our previous study, which focused on treatment-related changes in individual mediator concentrations [10], the present work examined the coordinated multivariate organization of inflammatory and regenerative responses within the same experimental system. Thus, the novelty of this reanalysis lies in evaluating how mediators are organized collectively and how these relationships vary according to treatment and time, rather than in generating new experimental observations.

Several limitations should be acknowledged. This in vitro model used acute LPS stimulation and may not fully reproduce the complexity of chronic osteoarthritis. In addition, only a selected panel of mediators was evaluated, and broader profiling could reveal additional dimensions of biological variability. Furthermore, principal component loadings describe patterns of covariation among mediators and should not be interpreted as evidence of causal biological relationships. Although the evaluated mediator ratios were selected a priori based on biological relevance, their multivariate organization may partly reflect the mathematical properties of ratio-based variables in addition to underlying biological processes. Although the number of biological donors was limited (n = 6), the repeated-measures design allowed within-donor comparisons across treatments and time points. Furthermore, bootstrap and leave-one-dog-out analyses supported the stability of the principal multivariate findings. Therefore, the results should be interpreted as evidence of multivariate organization within this controlled experimental system rather than as definitive population-level estimates. Accordingly, the evaluated mediator ratios should be regarded as complementary descriptors of coordinated biological responses rather than intrinsically superior biological markers, pending validation in independent experimental systems.

Despite these limitations, the present study demonstrates that multivariate analyses provide information that complements conventional univariate approaches and facilitates the characterization of coordinated mediator responses within complex orthobiologic systems. The findings support the incorporation of multivariate analytical frameworks into future studies evaluating platelet-derived secretomes and inflammatory microenvironments.

Accordingly, biologically relevant mediator ratios should be regarded as complementary descriptors of coordinated biological responses rather than intrinsically superior biological markers, pending validation in independent experimental and clinical studies.

## 3. Conclusions

This study demonstrates that cytokine, growth factor, and extracellular matrix-related mediator responses within an LPS-challenged osteoarthritis coculture system were distributed across multiple coordinated dimensions rather than being explained by individual biomarkers alone. Multivariate analyses identified distinct treatment- and time-related patterns involving combinations of inflammatory, regulatory, growth factor-related, and matrix-associated mediators.

The ratio-derived PCA concentrated a larger proportion of the total variance within fewer principal components and exhibited greater stability during bootstrap and leave-one-dog-out validation than the mediator-based PCA. These findings indicate that biologically relevant mediator ratios may provide a complementary multivariate perspective for characterizing coordinated mediator responses within this experimental system, although external validation is needed to confirm their broader applicability.

Among the evaluated treatments, PRGS 50% generated the most pronounced multivariate separation, particularly in components associated with growth factor- and matrix-related mediator profiles. However, these findings should be interpreted as evidence of greater biological activity within this experimental system rather than direct proof of clinical superiority.

Overall, the results support the use of multivariate analytical approaches as complementary tools to conventional univariate analyses for investigating the biological effects of platelet-derived secretomes and other orthobiologic interventions in complex inflammatory environments.

## 4. Materials and Methods

This study was approved by the Institutional Committee for Animal Experimentation (Authorization code: 0417118). The dogs included were classified as potentially dangerous under Colombian Law 746 of 2002 and Law 1801 of 2016 (National Code of Police and Coexistence) and had been previously retained by the competent authorities due to documented aggressive behavior. Euthanasia was performed under deep sedation followed by intravenous overdose of sodium phenobarbital, in accordance with the guidelines of the American Veterinary Medical Association (AVMA) Panel on Euthanasia [40]. All procedures were conducted in compliance with ARRIVE guidelines and applicable Colombian legislation governing animal welfare and research [41].

### 4.1. Study Design and Source of Data

This study represents a secondary integrative analysis of data generated in a controlled in vitro LPS-induced OA system evaluating the biological effects of PRGS and PPGS on cartilage–synovium explant cocultures. The original investigation focused on univariate mediator responses, and the complete experimental procedures (including tissue collection, hemocomponent preparation and activation, culture conditions, and biomolecule quantification) have been described previously [10]. No results, figures, or inferential conclusions from the prior study are reproduced herein.

The original dataset included biological material obtained from six clinically healthy dogs (n = 6; four females and two males; 1.5–3.5 years old; mean body weight 24.2 ± 2.87 kg), as previously described [10]. Hemocomponents and tissue explants were generated and applied in an autologous manner, such that each donor provided both the blood-derived products and the cartilage–synovium explants used throughout the experiment. Because all experimental conditions were repeated within each donor, the resulting dataset has a repeated-measures structure across treatment groups and time points. Each donor was considered one biological replicate, whereas ELISA determinations were performed in duplicate as technical replicates. In the present reanalysis, each dog was considered the primary experimental unit.

No new biological samples or experimental interventions were performed. Instead, the present work applies multivariate and mixed-effects analytical approaches to the existing dataset to characterize coordinated mediator organization, relative balance, and temporal dynamics within this biologically active system. This reanalysis was designed to evaluate relationships among multiple cytokines and GF that may not be evident through isolated univariate evaluation.

### 4.2. In Vitro Osteoarthritis System

Cartilage and synovial membrane explants were harvested under sterile conditions from canine stifle joints immediately following euthanasia and established in coculture under serum-free conditions. Explants were trimmed to standardized dimensions and equilibrated prior to stimulation. PRGS and PPGS were obtained from autologous whole blood collected from the same individual donors prior to euthanasia. PRP and plasma were generated by differential centrifugation and subsequently activated with calcium to induce gel formation [42]. Following clot retraction, cell-free supernatants (PRGS and PPGS) were recovered and stored under standardized conditions until use, as previously described [10]. Treatments were applied in an autologous manner, such that each explant coculture received hemocomponents derived from the same donor animal (Figure S1a–c).

The coculture system was challenged with lipopolysaccharide (LPS, 100 ng/mL) to induce inflammatory activation through TLR4-mediated signaling pathways [36]. Experimental conditions included a non-stimulated control, an LPS-stimulated control, and LPS-stimulated cultures treated with PRGS or PPGS at two concentrations, 25 and 50%. Culture media were collected at 1 h and 48 h following stimulation to assess early and later inflammatory and regulatory responses.

### 4.3. Biomarker Selection and Quantification

The reanalysis included IL-1β, IL-10, TGF-β1, PDGF-BB, and HA. These mediators were originally quantified in PRGS, PPGS, and coculture supernatants (1 h and 48 h) using enzyme-linked immunosorbent assay (ELISA). All measurements were performed in duplicate according to the manufacturer’s instructions.

Commercial ELISA development kits from R&D Systems (Minneapolis, MN, USA) were used. Canine-specific DuoSet kits were employed for IL-1β (DY3747) and IL-10 (DY735). HA concentrations were determined using the Hyaluronan DuoSet kit (DY3614), which allows multispecies detection. TGF-β1 (DY240E) and PDGF-BB (DY220) were measured using human-specific DuoSet kits due to the high sequence homology between human and canine proteins [43,44], as previously validated in canine PRP studies [10,42]. Standard curves were generated using the recombinant standards provided with each kit, and absorbance was read at 450 nm.

### 4.4. Statistical Analysis

All statistical analyses were performed in R (Version 4.5.2) (R Foundation for Statistical Computing, Vienna, Austria). Data processing and visualization were conducted using the packages readxl (v 1.4.5), dplyr (v 1.2.1), tidyr (v 1.3.2), ggplot2 (v 4.0.3), and tibble (v 3.3.0). Mixed-effects models were fitted using lme4 (v 1.1.38) and lmerTest (v 3.2.1), estimated marginal means were obtained with emmeans, correlation analyses were performed with Hmisc (v 5.2.5), and principal component analyses were conducted using FactoMineR (v 2.13) and factoextra (v 1.0.7) [45].

The dataset consisted of repeated measurements obtained at two time points for each donor and experimental condition. Time and experimental group were treated as categorical variables, and donor identity was included as a random effect to account for within-subject dependence [46].

The statistical workflow was designed to characterize the dataset at complementary analytical levels. Spearman correlation analysis was first used to explore pairwise associations among mediators. PCA was subsequently applied to identify coordinated multivariate patterns beyond pairwise relationships. Finally, linear mixed-effects models were used to evaluate treatment and time effects on the resulting multivariate structures while accounting for the repeated-measures design.

Associations among inflammatory and regenerative mediators were evaluated using Spearman rank correlations. To control for multiple testing, *p* values were adjusted using the Benjamini–Hochberg false discovery rate (FDR) procedure [47]. Correlation analyses were performed for the complete dataset and separately for each time point.

Mediator ratios were defined a priori based on biologically relevant relationships among inflammatory, regulatory, growth factor-related, and matrix-associated mediators rather than by statistical optimization. Consequently, only these predefined ratios were included in the multivariate analyses, and no data-driven ratio selection or optimization procedures were performed.

To characterize multivariate mediator organization, principal component analyses (PCA) were conducted using log-transformed mediator concentrations and biologically relevant log-transformed ratios. Separate PCA models were generated for mediator-derived and ratio-derived datasets. Principal component scores were extracted and subsequently analyzed using linear mixed-effects models, including treatment group, time, and their interaction as fixed effects, with donor identity specified as a random intercept [33]. Estimated marginal means and Holm-adjusted pairwise comparisons were calculated for significant effects.

The stability of multivariate findings was evaluated using bootstrap resampling (1000 iterations with replacement) and leave-one-dog-out analyses. Bootstrap resampling was used to estimate confidence intervals and sign stability for PCA loadings. For each iteration, PCA loadings were recalculated, and percentile confidence intervals and sign stability were estimated. Loading stability was evaluated by examining whether bootstrap confidence intervals crossed zero together with sign stability across iterations [48].

Leave-one-dog-out analysis consisted of repeating the complete analytical workflow after sequential exclusion of each donor (six iterations), allowing assessment of the influence of individual animals on principal component structure and mixed-model inference. Statistical significance was defined as a two-sided *p* value below 0.05.

**Figure 8:**
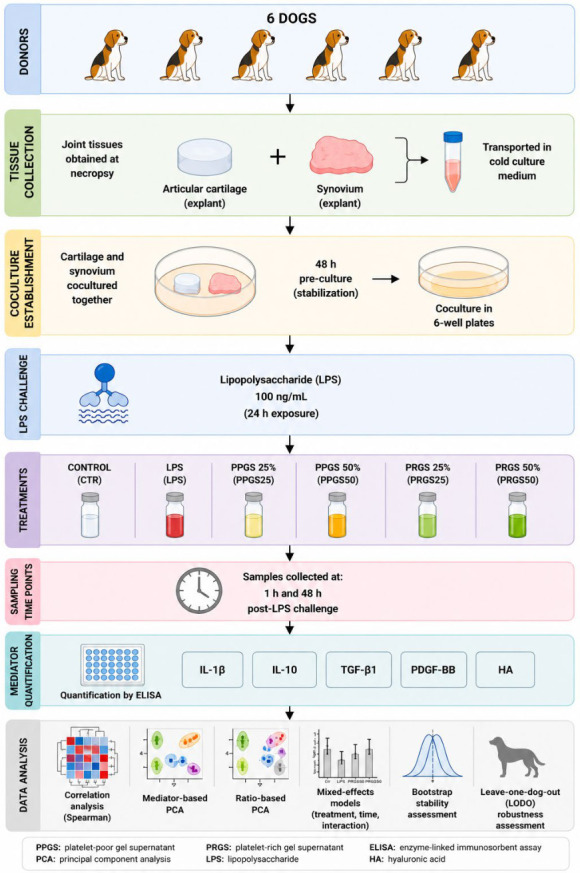
Schematic overview of the experimental design and analytical workflow. Abbreviations are as in Table 2.

The overall experimental design and analytical workflow are summarized in Figure 8, including tissue collection, coculture establishment, LPS challenge, treatment allocation, sampling time points, mediator quantification, and the subsequent multivariate statistical analyses performed in this study.

## Figures and Tables

**Table 1:** Baseline cellular characteristics of whole blood, platelet-rich plasma (PRP), and platelet-poor plasma (PPP) obtained from the six donor dogs (n = 6) before activation and preparation of platelet-rich gel supernatant (PRGS) and platelet-poor gel supernatant (PPGS).

Hemocomponent	WBC (×10^3^/µL) Mean ± SD	WBC Factor vs. WB	PLT (×10^3^/µL) Mean ± SD	PLT Factor vs. WB
Whole blood	11.48 ± 3.68	1.00	244.8 ± 30.5	1.00
PRP	1.00 ± 0.43	0.09	355.4 ± 39.5	1.45
PPP	0.18 ± 0.04	0.02	65.6 ± 17.5	0.27

Values are expressed as mean ± standard deviation (SD). Enrichment factors were calculated relative to the mean values of whole blood (WB = 1.00). Values > 1 indicate enrichment, whereas values < 1 indicate depletion relative to whole blood. WBC, white blood cells; PLT, platelets; PRP, platelet-rich plasma; PPP, platelet-poor plasma; WB, whole blood.

**Table 2:** Baseline molecular concentrations of platelet-rich gel supernatant (PRGS) and platelet-poor gel supernatant (PPGS) obtained from the same six donor dogs (n = 6).

Mediator	PPGS (Mean ± SD)	PRGS (Mean ± SD)	PRGS/PPGS Ratio
IL-1β (pg/mL)	18.45 ± 5.04	20.57 ± 5.93	1.11
IL-10 (pg/mL)	167.75 ± 96.00	174.51 ± 139.66	1.04
HA (ng/mL)	23.96 ± 25.91	25.16 ± 27.34	1.05
TGF-β1 (pg/mL)	83.20 ± 88.73	246.62 ± 96.52	2.96
PDGF-BB (pg/mL)	168.71 ± 87.01	1082.99 ± 367.22	6.42

Values are expressed as mean ± standard deviation (SD). The PRGS/PPGS ratio represents the relative enrichment of each mediator in PRGS compared with PPGS. PRGS, platelet-rich gel supernatant; PPGS, platelet-poor gel supernatant; IL-1β, interleukin-1 beta; IL-10, interleukin-10; HA, hyaluronic acid; TGF-β1, transforming growth factor beta 1; PDGF-BB, platelet-derived growth factor BB.

**Table 3:** Loadings of log-transformed inflammatory and regenerative mediators on the five principal components derived from the mediator-based principal component analysis (PCA) (n = 6).

Mediator	PC1	PC2	PC3	PC4	PC5
log IL-10	0.794	0.117	−0.074	−0.314	−0.501
log IL−1β	0.695	−0.425	−0.016	−0.333	0.475
log HA	0.448	0.622	−0.532	0.312	0.180
log TGF-β1	0.553	−0.536	0.195	0.599	−0.101
log PDGF-BB	0.317	0.695	0.630	0.032	0.136

Loadings indicate the contribution and direction of association of each log-transformed mediator with the corresponding principal component (PC). Positive and negative values indicate the direction of the association, whereas larger absolute loading values denote a greater contribution of the variable to the corresponding principal component. Other abbreviations are as in Table 2.

**Table 4:** Type III analysis of variance of linear mixed-effects models evaluating treatment, time, and treatment × time effects on mediator-derived principal components (n = 6).

PC	Effect	Sum Sq	Mean Sq	NumDF	DenDF	F Value	*p* Value
PC1	Group	33.052	6.610	5	66.00	6.447	<0.001
	Time	6.764	6.764	1	66.29	6.597	0.012
	Group × Time	3.418	0.684	5	66.27	0.667	0.650
PC2	Group	31.663	6.333	5	66.00	7.984	<0.001
	Time	1.381	1.381	1	66.33	1.741	0.192
	Group × Time	4.799	0.960	5	66.30	1.210	0.314
PC3	Group	3.828	0.766	5	66.00	1.780	0.129
	Time	7.636	7.636	1	66.16	17.748	<0.001
	Group × Time	3.202	0.640	5	66.15	1.488	0.206
PC4	Group	17.658	3.532	5	65.98	11.332	<0.001
	Time	0.010	0.010	1	66.20	0.032	0.859
	Group × Time	4.679	0.936	5	66.18	3.003	0.017
PC5	Group	8.462	1.692	5	66.00	6.151	<0.001
	Time	1.117	1.117	1	66.13	4.059	0.048
	Group × Time	3.870	0.774	5	66.12	2.813	0.023

Type III analysis of variance was performed on linear mixed-effects models fitted to the principal component (PC) scores, with treatment group, time, and their interaction as fixed effects and donor identity as a random effect. Sum Sq, sum of squares; Mean Sq, mean square; NumDF, numerator degrees of freedom; DenDF, denominator degrees of freedom. Other abbreviations are as in Table 3.

**Table 5:** Loadings of biologically relevant mediator ratios on the four principal components derived from the ratio-based principal component analysis (PCA) (n = 6).

Ratio	PC1	PC2	PC3	PC4
log HA/IL-10	0.936	−0.016	0.052	0.347
log IL-10/IL-1β	0.090	0.861	0.497	−0.062
log TGF-β1/IL-1β	0.004	0.864	−0.502	0.041
log PDGF-BB/HA	−0.932	0.070	0.098	0.343

Loadings indicate the contribution and direction of association of each log-transformed mediator ratio with the corresponding principal component (PC). Positive and negative values indicate the direction of the association, whereas larger absolute loading values denote a greater contribution of the ratio to the corresponding principal component. Other abbreviations are as in Table 2.

**Table 6:** Type III analysis of variance of linear mixed-effects models evaluating treatment, time, and treatment × time effects on ratio-derived principal components (n = 6).

PC	Effect	Sum Sq	Mean Sq	NumDF	DenDF	F Value	*p* Value
PC1	Group	7.056	1.411	5	66.00	1.447	0.219
	Time	14.259	14.259	1	66.09	14.620	<0.001
	Group × Time	4.530	0.906	5	66.08	0.468	0.798
PC2	Group	29.977	5.995	5	65.88	5.200	<0.001
	Time	7.469	7.469	1	66.35	6.000	0.017
	Group × Time	25.224	5.044	5	66.30	4.420	<0.001
PC3	Group	27.301	5.460	5	66.00	1.780	0.129
	Time	1.030	1.030	1	66.27	11.570	0.001
	Group × Time	0.926	0.185	5	66.25	2.079	0.079
PC4	Group	0.637	0.127	5	65.99	0.701	0.703
	Time	0.567	0.567	1	66.36	2.663	0.107
	Group × Time	0.483	0.097	5	66.32	0.453	0.810

Type III analysis of variance was performed on linear mixed-effects models fitted to the ratio-derived principal component (PC) scores, with treatment group, time, and their interaction as fixed effects and donor identity as a random effect. Sum Sq, sum of squares; Mean Sq, mean square; NumDF, numerator degrees of freedom; DenDF, denominator degrees of freedom. Other abbreviations are as in Table 2 and Table 3.

## Data Availability

The raw data supporting the conclusions of this article will be made available by the authors on request.

## Supplementary Materials

The following supporting information can be downloaded at https://www.mdpi.com/article/10.3390/gels12070615/s1; Figure S1: Representative images illustrating key stages of the experimental in vitro canine osteoarthritis coculture system. Table S1: Bootstrap stability statistics for mediator-derived and ratio-derived principal components. Table S2: Correlation between original and leave-one-dog-out scores. Table S3: Correlation between original and leave-one-dog-out scores.

## Abbreviations

The following abbreviations are used in this manuscript:

ANOVA	Analysis of variance
AUC	Area under the curve
CI	Confidence interval
EMM	Estimated marginal mean
FDR	False discovery rate
GF	Growth factor
HA	Hyaluronic acid
IL-1β	Interleukin-1 beta
IL-10	Interleukin-10
LMM	Linear mixed-effects model
LODO	Leave-one-dog-out
LPS	Lipopolysaccharide
NF-κB	Nuclear factor kappa B
OA	Osteoarthritis
PCA	Principal component analysis
PC	Principal component
PDGF-BB	Platelet-derived growth factor BB
PLT	Platelets
PPGS	Platelet-poor gel supernatant
PPP	Platelet-poor plasma
PRGS	Platelet-rich gel supernatant
PRP	Platelet-rich plasma
P-PRP	Pure platelet-rich plasma
ROC	Receiver operating characteristic
SD	Standard deviation
TGF-β1	Transforming growth factor beta 1
TLR4	Toll-like receptor 4
WB	Whole blood
WBC	White blood cells
