# Supplementary material for: Multivariate Analysis of Inflammatory and Regenerative Mediators in an In Vitro Canine Osteoarthritis System Treated with Platelet Gel Supernatants
Source: Gels. 2026 Jul 9;12(7):615. doi: 10.3390/gels12070615 (PMC13409436; doi:10.3390/gels12070615)
Supplement: Supplementary file 1 [file gels-12-00615-s001.zip › Supp Figure S1.pdf]

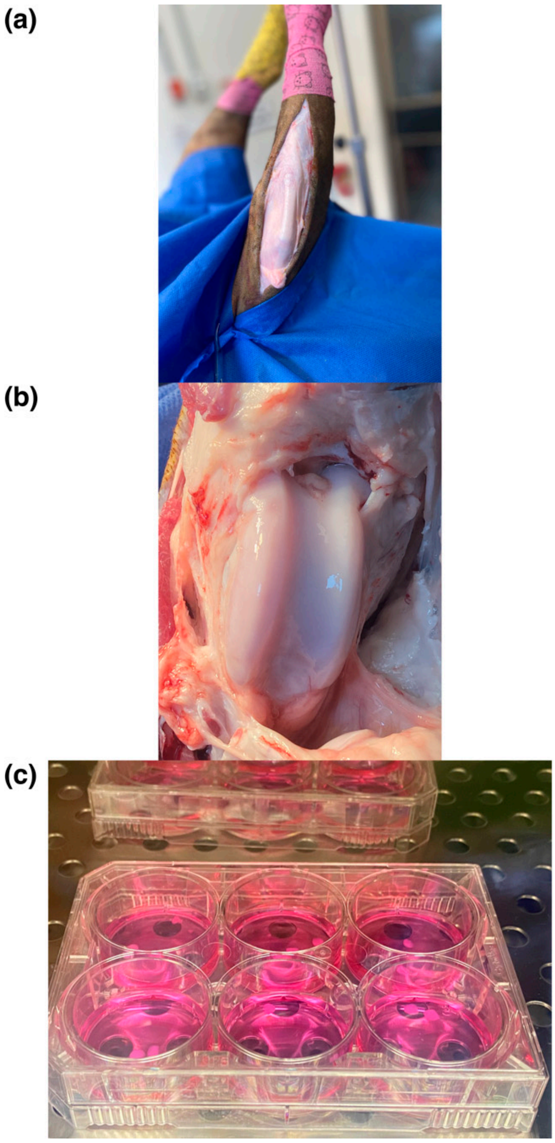

Figure S1. Representative images illustrating key stages of the experimental in vitro canine osteoarthritis coculture system. **(a)** Aseptically prepared surgical approach showing skin incision and exposure of the superficial joint tissues for collection of osteochondral and synovial explants. **(b)** Exposure of the articular surface during tissue collection immediately prior to explant harvesting. **(c)** Representative cartilage–synovium explants established in six-well culture plates for the in vitro coculture system. OA, osteoarthritis.
