# Supplementary material for: Multivariate Analysis of Inflammatory and Regenerative Mediators in an In Vitro Canine Osteoarthritis System Treated with Platelet Gel Supernatants
Source: Gels. 2026 Jul 9;12(7):615. doi: 10.3390/gels12070615 (PMC13409436; doi:10.3390/gels12070615)
Supplement: Supplementary file 1 [file gels-12-00615-s001.zip › Tables S1-S3.pdf]

Table S1. Bootstrap stability statistics for mediator-derived and ratio-derived principal components.

PCA type	Variable	PC	Mean loading	SD loading	Lower 95% CI	Upper 95% CI	Sign stability
Mediator PCA	HA	PC1	0.214	0.439	-0.782	0.699	0.781
	HA	PC2	0.546	0.261	-0.276	0.828	0.942
	HA	PC3	-0.394	0.256	-0.753	-0.193	0.909
	HA	PC4	0.150	0.301	0.438	0.594	0.667
	HA	PC5	0.143	0.206	-0.331	0.484	0.786
	IL-10	PC1	0.567	0.486	-0.777	0.869	0.862
	IL-10	PC2	0.102	0.307	-0.732	0.779	0.696
	IL-10	PC3	-0.051	0.276	-0.553	0.472	0.547
	IL-10	PC4	-0.156	0.258	-0.574	0.429	0.742
	IL-10	PC5	-0.389	0.132	-0.551	0.039	0.971
	IL-1β	PC1	0.624	0.281	-0.301	0.848	0.928
	IL-1β	PC2	-0.359	0.262	-0.809	0.253	0.917
	IL-1β	PC3	0.005	0.277	-0.524	0.530	0.508
	IL-1β	PC4	-0.255	0.238	-0.604	0.337	0.857
	IL-1β	PC5	0.329	0.169	-0.143	0.536	0.934
	PDGF-BB	PC1	0.070	0.447	-0.767	0.673	0.625
	PDGF-BB	PC2	0.579	0.241	-0.142	0.844	0.955
	PDGF-BB	PC3	0.462	0.190	0.016	0.735	0.979
	PDGF-BB	PC4	0.015	0.325	-0.553	0.562	0.510
	PDGF-BB	PC5	0.096	0.195	-0.287	0.449	0.700
	TGF-β1	PC1	0.521	0.248	-0.246	0.784	0.940
	TGF-β1	PC2	-0.452	0.254	-0.815	0.153	0.945
	TGF-β1	PC3	0.405	0.373	-0.580	0.713	0.647
	TGF-β1	PC4	0.389	0.197	-0.099	0.650	0.943
	TGF-β1	PC5	-0.039	0.217	-0.413	0.405	0.597
Ratio PCA	HA/IL-10	PC1	0.876	0.058	0.750	0.948	1.000
	HA/IL-10	PC2	-0.016	0.323	-0.492	0.513	0.520
	HA/IL-10	PC3	0.054	0.093	0.121	0.237	0.744
	HA/IL-10	PC4	0.335	0.036	0.269	0.412	1.000
	IL-10/IL-1β	PC1	0.083	0.396	-0.583	0.679	0.558
	IL-10/IL-1β	PC2	0.762	0.092	0.556	0.884	0.999
	IL-10/IL-1β	PC3	0.485	0.054	0.381	0.576	1.000
	IL-10/IL-1β	PC4	-0.064	0.069	-0.211	0.055	0.841
	PDGF-BB/HA	PC1	-0.862	0.071	-0.947	-0.711	1.000
	PDGF-BB/HA	PC2	0.065	0.035	-0.474	0.583	0.545
	PDGF-BB/HA	PC3	0.100	0.093	-0.085	0.278	0.871
	PDGF-BB/HA	PC4	0.329	0.039	0.260	0.403	1.000
	TGF-β1/IL-1β	PC1	0.006	0.395	-0.637	0.621	0.502
	TGF-β1/IL-1β	PC2	0.765	0.087	0.571	0.887	1.000
	TGF-β1/IL-1β	PC3	-0.493	0.051	-0.588	-0.394	1.000
	TGF-β1/IL-1β	PC4	0.005	0.051	-0.037	0.397	0.717

Sign stability represents the proportion of bootstrap iterations retaining the original loading sign. CI, confidence interval; PC, principal component; SD, standard deviation. Sign stability represents the proportion of bootstrap iterations retaining the original loading sign. HA, hyaluronic acid; IL-10, interleukin-10; IL-1β, interleukin-1 beta; TGF-β1, transforming growth factor beta 1; PDGF-BB, platelet-derived growth factor-BB.

Table S2. Correlation between original and leave-one-dog-out scores.

**PCA type**	PC	Mean correlation	SD	Minimum	Maximum
Mediator PCA	PC1	0.984	0.020	0.947	0.999
Mediator PCA	PC2	0.959	0.052	0.868	0.999
Mediator PCA	PC3	0.741	0.278	0.373	0.978
Mediator PCA	PC4	0.870	0.107	0.673	0.958
Mediator PCA	PC5	0.825	0.344	0.122	0.992
Ratio PCA	PC1	0.864	0.179	0.507	0.999
Ratio PCA	PC2	0.958	0.068	0.822	0.999
Ratio PCA	PC3	0.995	0.006	0.982	0.999
Ratio PCA	PC4	0.990	0.011	0.969	0.999

Abbreviations as in Tables S1.

Table S3. Correlation between original and leave-one-dog-out scores.

PCA type	PC	Effect	Mean F	SD F	Median p	Minimum p	Maximum p	Significant iterations (%)
Mediator PCA	PC1	Group	5.70	4.76	0.0004	<0.0001	0.803	66.7
Mediator PCA	PC1	Time	4.90	2.00	0.036	0.0007	0.138	83.3
Mediator PCA	PC1	Group × Time	0.64	0.29	0.736	0.384	0.899	0.0
Mediator PCA	PC2	Group	10.85	9.26	<0.0001	<0.0001	0.096	83.3
Mediator PCA	PC2	Time	1.75	1.53	0.358	0.001	0.701	33.3
Mediator PCA	PC2	Group × Time	7.96	15.94	0.233	<0.0001	0.923	16.7
Mediator PCA	PC3	Group	3.14	3.17	0.153	<0.0001	0.475	50.0
Mediator PCA	PC3	Time	1.71	0.68	0.193	0.033	0.524	16.7
Mediator PCA	PC3	Group × Time	13.29	7.81	0.002	<0.0001	0.014	100.0
Mediator PCA	PC4	Group	10.72	3.76	<0.0001	<0.0001	<0.001	100.0
Mediator PCA	PC4	Time	3.76	3.15	0.035	<0.0001	0.271	66.7
Mediator PCA	PC4	Group × Time	4.29	3.82	0.123	0.003	0.525	50.0
Mediator PCA	PC5	Group	4.31	2.03	0.002	<0.001	0.464	83.3
Mediator PCA	PC5	Time	2.15	1.04	0.103	0.008	0.614	33.3
Mediator PCA	PC5	Group × Time	3.15	4.14	0.202	0.001	0.540	16.7
Ratio PCA	PC1	Group	2.00	0.96	0.174	0.012	0.384	33.3
Ratio PCA	PC1	Time	1.65	1.24	0.243	0.004	0.886	16.7
Ratio PCA	PC1	Group × Time	15.05	11.34	<0.001	<0.0001	0.469	83.3
Ratio PCA	PC2	Group	8.43	3.76	<0.0001	<0.0001	<0.001	100.0
Ratio PCA	PC2	Time	7.56	5.27	<0.001	<0.0001	0.008	100.0
Ratio PCA	PC2	Group × Time	11.50	7.09	0.001	<0.0001	0.818	83.3
Ratio PCA	PC3	Group	53.75	10.18	<0.0001	<0.0001	<0.0001	100.0
Ratio PCA	PC3	Time	2.20	1.23	0.122	0.001	0.357	16.7
Ratio PCA	PC3	Group × Time	10.64	2.84	0.002	<0.001	0.016	100.0
Ratio PCA	PC4	Group	0.75	0.59	0.671	0.145	0.963	0.0
Ratio PCA	PC4	Time	0.55	0.29	0.715	0.452	0.981	0.0
Ratio PCA	PC4	Group × Time	2.48	1.67	0.161	0.020	0.980	16.7

LODO, leave-one-dog-out. Significant iterations (%) indicates the proportion of leave-one-dog-out iterations in which the corresponding effect remained statistically significant ($p < 0.05$). Abbreviations as in Tables S1.
